# Demographic, epidemiological and clinical profile of patients with post-COVID syndrome followed at a teaching hospital in Brazil

**DOI:** 10.1016/j.bjid.2025.104509

**Published:** 2025-02-19

**Authors:** João Paulo Borges de Melo, Alex Eduardo da Silva, Leandro Resende Yamamoto, Taciana Fernandes Araújo Ferreira, Gustavo José Luvizutto, Fernando Freitas Neves, Kelly Cristina Santos, Roger Lopes Batista, Isabel Cunha Santos, Francielle Schiavoni, Mario León Silva-Vergara

**Affiliations:** Universidade Federal do Triângulo Mineiro, Departamento de Medicina Interna, Departamento de Fisioterapia, Uberaba, MG, Brazil

**Keywords:** COVID-19, Post-COVID syndrome, Chronic fatigue, Brain fog, SARS-CoV-2

## Abstract

**Introduction:**

Post-COVID Syndrome (PCS), occurs several weeks after Severe Acute Respiratory Syndrome Coronavirus (SARS-CoV-2 infection), has a frequency of 10 %‒35 % of cases, presents a wide range of symptoms that can persist for months or years and markedly reduces the quality of life of patients.

**Objective:**

To describe clinical, epidemiological and evolutionary aspects of a cohort of patients diagnosed with PCS followed on an outpatient basis.

**Methodology:**

Individuals of both sexes, > 18-years old who presented symptoms suggestive of PCS and had previously confirmed SARS-CoV-2 infection were included. Clinical evaluation was carried out monthly by a multidisciplinary team, and if necessary laboratorial exams were performed.

**Results:**

From June 2021 to June 2022, 92 cases of PCS were diagnosed, of which 60 (65.2 %) were female and the average age was 49.1 years. In 61 (66.3 %) of the cases, SARS-CoV-2 infection occurred between January and November 2021. In 55 (59.7 %) of the cases the symptoms were mild, while 31 (36.0 %) were moderate or severe cases. Most cases of PCS occurred in individuals with the mild form of COVID-19. The predominant symptoms were chronic fatigue in 59 (68.6 %) cases, brain fog in 68 (73.4 %), myalgias and arthralgias in 44 (47.8 %), cramps and paresthesia's in 40 (46.5 %). The main comorbidities observed were high blood pressure, obesity and diabetes mellitus. The persistence of symptoms was greater in those cases who presented severe forms of COVID-19. Most patients experienced gradual and progressive improvement over the months.

**Discussion:**

The profile of patients with PCS in this cohort is similar to other reports. A great number of symptoms is remarkable with variable presentation and evolution and their frequency exceeds that previously described in a large meta-analysis. Inflammatory phenomena mediated by the virus, autoimmunity and direct organic damage have been implicated in the genesis of this syndrome.

## Introduction

At the end of 2019, SARS-CoV-2 infection, spread globally and was declared at pandemic level by the World Health Organization (WHO) in March 2020. Infection can range from asymptomatic until acute respiratory distress syndrome with multiple organ dysfunction and high rates of unfavorable outcome.^1^^,^^2^ Recent data from the WHO showed the occurrence of around 770 million confirmed cases of COVID-19, with almost 13 million deaths and >13 billion doses of vaccines administered. However, these estimates and according to several authors, may be numerically higher.^3^

Since the beginning of the pandemic, it was evidenced that 10 % to 35 % of individuals with acute SARS-CoV-2 infection presented new or persistent symptoms after the acute phase, related to different organs and systems.^4^, ^5^, ^6^ In September 2020, the WHO included this new health condition in the International Classification of Diseases (ICD), under the term “health condition subsequent to COVID-19 – U09”.^7^

Several nomenclatures have emerged to name this new clinical condition, such as long COVID-19, long-lasting or long-distance (“long haul”), in addition to chronic COVID-19 syndrome, late sequelae of COVID-19, Post-COVID Syndrome (SPC), post-acute COVID-19 and post-acute sequelae of SARS-CoV-2 infection.^5^^,^^8^^,^^9^

The relationship between the clinical forms of COVID-19 and the risk of developing PCS was established by several authors who observed greater frequency and severity among those patients who presented severe clinical forms of the disease and underlying comorbidities. To date, around 200 different symptoms associated with PCS have been described and the most prevalent can be subdivided into physical, cognitive and psychiatric.

Of the physical symptoms associated to SPC, the main ones are chronic fatigue 17 % to 30 % of patients, myalgias and arthralgias 4 %‒22 %; dyspnea 11 % to 15 %; changes in smell and taste 6 %‒23 %.^10^^,^^11^ Regarding cognitive symptoms: brain fog 10 %‒19 %.^12^^,^^13^ Among the psychiatric disorders, the main ones are depression, anxiety, 3 %‒25 % and sleep disorders 5 %‒23 %.^12^, ^13^, ^14^

The occurrence and persistence of these symptoms varies greatly over time and their genesis has been attributed to several factors such as the inflammatory process mediated by the virus, autoimmunity phenomena and direct organic damage, among others.^15^^,^^16^ The high frequency of PCS and its impact on patients’ functionality, independence and quality of life is very relevant and need to be more evaluated. Furthermore, the therapeutic and rehabilitation difficulties together with the high medical, economic and social burden due to these patients' work and school absenteeism require special attention as they currently represent a global public health problem.^3^^,^^17^, ^18^, ^19^

The present report aims to describe the demographic, epidemiological and clinical- evolutionary profile of a cohort of patients with PCS, treated in an outpatient service of a tertiary level university hospital in Brazil.

## Methods

This prospective observational cohort study was carried out at the post-COVID outpatient clinic of the Hospital de Clinicas of the *Universidade Federal do Triângulo Mineiro* from June 2021 to June 2022. For this study, the term Post-COVID Syndrome (PCS), as a synonym for the clinical conditions included in ICD-10 U09.WHO.^7^ To this end, patients with new and persistent symptoms after 4-weeks of the acute phase of COVID-19, with no other etiology defined and previously non-existent were included.

All patients > 18-years old, of both sexes, with a clinical condition compatible with PCS, were gradually registered and invited to participate in the study and sign the free and informed consent form. Demographic, epidemiological and clinical evolutionary data were obtained throughout the outpatient follow-up, which lasted until substantial improvement and/or complete resolution of symptoms. The clinical categories of SARS- CoV-2 infection were defined based on patient information: asymptomatic case; mild case when there were general symptoms, but did not need oxygen support and he remained at home; moderate case when required hospitalization and non-invasive respiratory support and severe case when underwent orotracheal intubation and intensive care in a specialized unit was necessary.

At each monthly consultation, patients were evaluated by a multidisciplinary team made up of an infectious disease specialist, a pulmonologist and a neurologist, as well as a physiotherapist, psychologist, nutritionist and a social worker. Depending on the clinical symptoms presented, functional tests and complementary exams, were performed. Patients with osteoarticular and respiratory symptoms were referred to specific physiotherapy.

Patients with confirmed SARS-CoV-2 reinfection during follow-up were excluded from the cohort. Of the 92 cases that remained, 36 (39.1 %) did not return for follow-up at the second consultation. This sample reduction occurred gradually during follow-up and the number of new patients decreased drastically from January 2022 onwards.

The data obtained were analyzed using descriptive statistics and the chi-square test and Fisher's exact test were applied when indicated. Values < 0.05 were considered statistically significant. The present study was approved by the Ethics Committee of the *Universidade Federal do Triângulo Mineiro*.

## Results

Of the 92 patients initially evaluated, 60 (65.2 %) were female. The average age was 49.1 years. The majority had completed secondary or higher education and reported very diverse occupations. The most prevalent comorbidities were arterial hypertension 36 (39.1 %) cases, obesity 27 (29.3 %) and diabetes mellitus 21 (22.8 %). Regarding lifestyle habits, 9 (9.8 %) had a history of smoking, 22 (23.9 %) drank alcoholic beverages and 18 (19.5 %) reported practicing physical activities regularly.

The majority, 61 (66.3 %), of patients received a diagnosis of acute SARS-CoV-2 infection between January and November 2021. In 28 (30.4 %) of cases, the diagnosis was made in Basic Health Units. In 20 (21.7 %) in Reference Hospitals, in 10 (10.8 %) in Emergency Care Units and 34 (37.0 %) in private laboratories. Diagnostic confirmation was made using a rapid nasal swab test in 57 (62.0 %) of the cases and by PCR in 35 (38.0 %). The main motivation for seeking a diagnosis was the presence of flu syndrome in 50 (54.3 %) of the cases, followed by contact with a confirmed or suspected case in 42 (45.6 %) individuals.

The main symptoms were chronic fatigue in 59 (64.1 %) cases, brain fog in 68 (74.3 %), myalgias and arthralgias in 44 (47.8 %) and cramps and paresthesia's in 40 (43.5 %). The distribution of these symptoms according to the clinical form of acute SARS-CoV-2 infection showed a predominance in those who had the mild form of COVID-19 (Table 1, Table 2).

**Table 1:** Main symptoms referred by 92 patients with Post COVID Syndrome.

Symptoms	n	%
Chronic fatigue	59	64.1
Myalgias e arthralgias	44	47.8
Alopecia	30	32.6
Cuff	27	29.3
Shorness of breath	27	29.3
Tachycardia/chest pain	32	34.8
Loss of the sense of smell	27	29.3
Headache	26	28.2
Vertigo	32	34.8
Cramps/Paresthesias	40	43.5
Brain fog	68	73.4
Sleep disorders	39	42.4
Mood disorders	30	32.6

**Table 2:** Main symptoms in patients with Post COVID Syndrome according to the clinical picture of COVID-19.

Post COVID symptoms						
Patients and clinical Picture of COVID-19		Cuff and dyspnea	Chronic fatigue	Myalgias and arthralgias	Brain fog	Tachycardia / Chest pain
	n (%)	n (%)	n (%)	n (%)	n (%)	n (%)
Mild	55 (64.0)	27 (49.1)	30 (54.5)	23 (41.8)	38 (69.1)	16 (29.1)
Moderate	18 (21.0)	14 (77.8)	16 (88.9)	11 (61.1)	17 (94.4)	12 (66.6)
Severe	13 (15.1)	13 (100.0)	13 (100.0)	10 (76,9)	13 (100.0)	4 (30.7)
Total		54-	59-	44-	68-	32-

The laboratory changes found in these patients were mainly tests of inflammatory activity, blood count, blood glucose and d-dimer. Among the imaging/functional tests, spirometry and echocardiogram were altered in a substantial number of cases, as described in Table 3, Table 4.

**Table 3:** Laboratorial assessment of patients with Post COVID Syndrome.

Test	Abnormal		Normal		Total
	n	%	n	%	
Blood count	20	36.4	35	63.6	55
PCR and ERS	23	48.0	25	52.1	48
D-dimer	13	33.3	26	66.6	39
TSH and T4L	5	10.6	42	89.3	47
Seric urea and creatinine	9	18.0	41	82.0	50
ALT and AST	4	8.7	42	91.3	46
Glicose e Hb1Ac	17	34.7	32	65.3	49
Vitamin D	11	36.7	19	63.3	30
Vitamin B12	1	3.3	29	96.6	30
Folinic acid	3	13.0	20	86.9	23

**Table 4:** Radiological assessment and functional tests in patients with Post COVID syndrome.

Test	Abnormal	Normal	Patients
	n (%)	n (%)	n (%)
Chest tomography	17 (70.8)	7 (29.2)	24
Skull tomography	8 (61.5)	5 (38.5)	13
Echocardiogram	28 (54.9)	23 (45.1)	51
Spirometry	17 (37.0)	29 (63.0)	46

During outpatient follow-up, the patients showed progressive improvement in the symptoms, although some still remained symptomatic in the sixth month. There was a gradual sample reduction during follow-up, such that in the second consultation 56 (60.9 %) patients attended, in the third 31 (33.7 %) patients, in the fourth 23 (25.0 %), in the fifth 19 (20.6 %) and only 9 (9.8 %) patients in the sixth month (Table 5). The persistence of symptoms was greater in those cases who presented severe forms of COVID-19.

**Table 5:** Evolution of symptoms in patients with Post COVID Syndrome during the follow-up.

Symptoms	Monthly follow-up				
	2	3	4	5	6
	n (%)	n (%)	n (%)	n (%)	n (%)
Cuff and dyspnea	23 (41.1)	16 (51.6)	15 (65.2)	11 (57.9)	6 (66.6)
Chronic fatigue	15 (26.8)	9 (29.0)	13 (56.5)	8 (42.1)	4 (44.4)
Myalgias and arthralgias	20 (35.7)	9 (29.0)	10 (43.5)	7 (36.8)	2 (22.2)
Brain fog	14 (25.0)	4 (12.9)	4 (17.4)	6 (31.5)	2 (22.2)
Tachicardia/Chest pain	11 (19.6)	7 (22.6)	7 (30.4)	7 (36.8)	2 (22.2)
Patients	56	31	23	19	9

## Discussion

Recently, the definition of PCS was revised according to more comprehensive and homogeneous criteria that can allow a more holistic clinical approach, in addition to facilitating comparisons between different studies. So, it was defined.as “an infection-associated chronic condition that occurs after SARS-CoV-2 infection and is present for at least 3-months as an ongoing, relapsing-remitting, or progressive disease state that affects one or more organ or systems”.^20^

Most current conservative global estimates point to 65 million individuals with PCS, a number that continues to grow according to several authors. The incidence of PCS is variable and ranges from 10 %‒30 % in patients not hospitalized for COVID-19, 50 %‒70 % among those presenting severe disease that required hospitalization and 10 %‒12 % of cases among those who were vaccinated. However, PCS can occur in all age groups, although the majority of cases described presented a mild clinical form which represents the largest number of cases.^21^, ^22^, ^23^

The majority had presented acute infection and symptoms of PCS before December 2021, when the Omicron variants began to be described and were not yet circulating.in Brazil. After this date, the number of acute infections increased, but severity and mortality decreased substantially.^21^, ^22^, ^23^ Furthermore, it was suggested that as the population reached high vaccination rates, the occurrence of acute infection and consequently PCS would also have decreased.^24^^,^^25^

O risk of PCS is increased in individuals with severe COVID-19 and preexisting comorbidities A recent report shown the cumulative incidence of PCS at 1-year after infection decreased during the pandemic from 10.42 cases per 100 persons among unvaccinated individuals during the pre-delta era to 3.50 per 100 persons among vaccinated individuals during the omicron era.^26^

Since the first cases described a correlation between the severity of the acute phase of COVID-19 and the occurrence of PCS was evidenced.^6^ Several authors have reported a higher prevalence of PCS in patients who had severe COVID-19,^5^^,^^10^^,^^11^ although it can occur regardless of the intensity of the acute clinical form or whether it was asymptomatic. On the other hand, the presence of previous lung diseases, smoking habit, female gender, obesity and age > 65-years were also factors associated with a higher risk of developing PCS.^22^^,^^23^^,^^27^

A multi-database study with two large cohorts showed that patients with severe COVID-19, > 65-years-old, presence of several comorbidities and incomplete vaccination had a much higher risk of having PCS with much more varied and severe symptoms.^21^ In the present cohort, arterial hypertension was evident in 36 (39.1 %) cases, obesity in 27 (29.3 %) and diabetes mellitus in 21 (22.8 %) as described by others. Although, 61 % of cases of SPC had mild symptoms during the SARS-CoV-2 infection, the highest frequency of symptoms was seen in those patients who presented severe or critical COVID-19, which is in line with other studies (Table 2).^21^^,^^22^^,^^28^

Symptoms such as chronic fatigue, brain fog, muscular and articular pain and respiratory complaints as reported by several authors were also confirmed in the cohort of patients herein reported.^29^^,^^30^ However, the frequency was higher than previously described.^14^ Chronic fatigue occurred in 64 % of the 92 patients evaluated and it is one of the most prevalent and persistent symptom in the different case series already published.

Since the first reports of PCS, this symptom has drawn attention due to its similarity with Chronic Fatigue Syndrome (CFS), or myalgia encephalomyelitis, which presents chronic fatigue, sleep disorders, neurocognitive changes, associated with diverse autonomic disorders. CFS, in turn, has an obscure pathophysiology, and has been associated with viral infections, such as Epsten-Bar, Cytomegalovirus and Herpes virus, among others, which analogously could also be related to acute infection by SARS-CoV-2.^31^, ^32^, ^33^, ^34^, ^35^ This can be corroborated by data from the literature that showed the development of a similar picture after the occurrence of epidemics associated with other coronaviruses such as SARS in 2003 and MERS in 2012.^29^^,^^30^

During the follow-up of these patients in the sixth month and although fewer in number, 66 % of cases persisted with respiratory complaints and 44 % with chronic fatigue (Table 5). The duration of PCS symptoms is still uncertain and very variable from patient to patient and, according to different authors, it can last for months or years and be influenced by previous comorbidities, as well as the severity of the COVID-19 disease.^34^, ^35^, ^36^, ^37^, ^38^, ^39^ In general, neurological and psychiatric changes can persist beyond six months in up to 33 % of cases and predominantly in females.^27^^,^^37^, ^38^, ^39^

Several hypotheses have been raised to explain the pathophysiology of PCS symptoms, such as inflammatory phenomena mediated by different cytoquines due to viral persistence, autoimmunity, genetic predisposition, presence of microthrombi and endothelial dysfunction in different organs, and in the case of patients who had a severe acute illness, sequelae of critical illness – Post-Intensive Care Syndrome.^31^, ^32^, ^33^, ^34^, ^35^, ^36^ Since its first descriptions, PCS has been considered an extremely complex disease in its pathophysiological, clinical, evolutionary and socioeconomic contexts.^21^^,^^22^

Although they were not quantified in the present study, work and school absenteeism and compromised quality of life due to the persistence of symptoms were very frequent in patients with PCS. The continuous prescription of various medications to alleviate multiple symptoms, combined with the constant requirement for medical certificates, and the need for respiratory, musculoskeletal and psychological rehabilitation therapies, reinforce these facts. Several authors who have addressed these topics confirm these observations. In this sense, it was described that the greater the number and/or severity of PCS symptoms, the greater the negative impact on quality of life, independence, physical capacity, and work and school attendance in these patients.^40^, ^41^, ^42^, ^43^, ^44^ Thus, these topics require special attention as they currently represent a global public health problem, besides a medical, economic and social high burden.^3^^,^^17^, ^18^, ^19^

## Conflicts of interest

The authors declare no conflicts of interest.
